# QTL Mapping of Kernel Number-Related Traits and Validation of One Major QTL for Ear Length in Maize

**DOI:** 10.1371/journal.pone.0155506

**Published:** 2016-05-13

**Authors:** Dongao Huo, Qiang Ning, Xiaomeng Shen, Lei Liu, Zuxin Zhang

**Affiliations:** 1 National Key Laboratory of Crop Genetic Improvement, Huazhong Agricultural University, Wuhan 430070, P. R. China; 2 Hubei Collaborative Innovation Center for Grain Crops, Jingzhou 434025, P. R. China; Institute of Genetics and Developmental Biology, CHINA

## Abstract

The kernel number is a grain yield component and an important maize breeding goal. Ear length, kernel number per row and ear row number are highly correlated with the kernel number per ear, which eventually determines the ear weight and grain yield. In this study, two sets of F_2:3_ families developed from two bi-parental crosses sharing one inbred line were used to identify quantitative trait loci (QTL) for four kernel number-related traits: ear length, kernel number per row, ear row number and ear weight. A total of 39 QTLs for the four traits were identified in the two populations. The phenotypic variance explained by a single QTL ranged from 0.4% to 29.5%. Additionally, 14 overlapping QTLs formed 5 QTL clusters on chromosomes 1, 4, 5, 7, and 10. Intriguingly, six QTLs for ear length and kernel number per row overlapped in a region on chromosome 1. This region was designated *qEL1*.*10* and was validated as being simultaneously responsible for ear length, kernel number per row and ear weight in a near isogenic line-derived population, suggesting that *qEL1*.*10* was a pleiotropic QTL with large effects. Furthermore, the performance of hybrids generated by crossing 6 elite inbred lines with two near isogenic lines at *qEL1*.*10* showed the breeding value of *qEL1*.*10* for the improvement of the kernel number and grain yield of maize hybrids. This study provides a basis for further fine mapping, molecular marker-aided breeding and functional studies of kernel number-related traits in maize.

## Introduction

Maize is one of the most widely grown crops worldwide. The rapidly expanding global demand for maize as a food, feed and industrial crop has led to intense pressure to improve the maize grain yield, which is an extremely complex quantitative trait controlled by quantitative trait loci (QTLs)[1–3]. The genetic complexity and low heritability impede our understanding of the genetic basis and molecular mechanisms underlying grain yield. The maize grain yield is composed of yield components that include kernel number per ear (KN) and kernel weight (KW). Kernel number is composed of the ear row number (ERN) and kernel number per row (KNR). The yield components exhibit higher heritability and better stability across environments compared with the grain yield[2,4]. Generally, yield components (ERN, KNR and KW) are highly positively correlated with the grain yield[5,6]. Therefore, the identification and isolation of QTLs for yield components instead of the grain yield itself would be more effective for dissecting the genetic basis and guiding the genetic improvement of the maize grain yield.

Since the first molecular marker linkage map of maize was published in 1986 [7], hundreds of QTLs for yield and yield-related traits have been identified on the maize genome through linkage mapping [1,2,4,8]. These increasing QTL data have expanded our knowledge of the genetic basis of yield and yield-related traits. Another alternative strategy to uncover natural variations in complex traits is the genome-wide association study (GWAS), which has become an obvious general approach to study the genetic architecture of agriculturally important crop plant traits [9–11]. Genome-wide nested association mapping can improve the power and the accuracy of QTL detection and identify desirable alleles relative to the founder and the lines harboring the desirable alleles. By combining linkage and association mapping, Liu et al. (2015) [12] identified 17 KRN-associated genomic loci in an association panel composed of 513 inbred lines and 21 common KRN QTLs in three linkage populations and suggested that the maize kernel row number might be dominated by a set of large additive or partially dominant loci and several small dominant loci. Brown et al. (2011) [13] identified 236 joint linkage QTLs and 1,966 GWAS SNPs for seven inflorescence architecture-related traits, such as kernel row number, cob diameter, and tassel length. Additionally, these authors found that a few of the cloned inflorescence mutants in maize were co-localized with GWAS SNPs and that the majority of the loci controlling natural variation in maize inflorescence traits were distinct from the genes detected using mutants. The results show that GWAS analysis can detect more new loci that are significantly associated with complex traits.

The dissection of the genetic basis of grain yield eventually depends on the isolation of genes underlying the QTL or the association loci for grain yield-related traits. However, it is difficult to isolate the genes for either association loci or QTLs because these loci mostly map to a large chromosome interval. Therefore, it is essential to validate and narrow down their positions for map-based cloning and marker-aided backcrossing. To date, only a few of the QTLs for grain yield-related traits have been isolated, including *fasciated ear2* (*fea2*) [14], *unbranched3* (*Ub3*) [15] and *KRN4* [16] for kernel row number. The limited number of cloned genes has led to a poor understanding of the molecular regulation of the grain yield in maize.

In the present study, we attempt to: i) identify QTLs for the kernel number-related traits ear row number, kernel number per row, ear length and ear weight in maize using two mapping populations derived from two bi-parental crosses sharing a parental line and ii) to validate and narrow down a major QTL for kernel number and ear length in an advanced backcross population and to evaluate the breeding value for the improvement of grain yield. This study will provide a basis for further fine mapping, molecular marker-aided breeding and functional studies of grain yield-related traits in maize.

## Materials and Methods

### Plant materials

Three elite inbred lines [Mo17 (a public inbred line related to Lancaster Sure Crop), TY6 (a inbred line-derived from Wu109 ×Huangzao4) and W138 (a Dan340-derived line)] were used to develop two bi-parental populations: Mo17×TY6 (designated MT hereafter) and W138×TY6 (designated WT hereafter). One hundred ninety F_2_ individuals from the MT and 269 F_2_ individuals from the WT populations were randomly chosen to generate two sets of F_2:3_ families through selfing. A whole genome scan for QTLs was conducted in the two sets of F_2:3_ families. *qEL1*.*10* was a major QTL for EL identified repeatedly in the two sets of F_2:3_ families. To validate this QTL, marker-aided backcrossing was performed using W138 as the recurrent parent and TY6 as the donor. A BC_4_F_1_ individual with a heterozygous marker genotype within the *qEL1*.*10* interval was chosen and then selfed to develop a homozygous QTL near-isogenic line (QTL-NIL) carrying an allele from TY6 at *qEL1*.*10* (designated W138^TY6^). Then, W138^TY6^ was crossed with W138 and selfed to develop an NIL-derived population with 235 F_3_ families.

### Field experiment and trait evaluation

Field experiments were performed in the Xingtai Institute of Agricultural Science and the Huanggang Institute of Agricultural Science. No specific permissions were required for performing these field experiments, and the field studies did not involve endangered or protected species.

The two sets of F_2:3_ families and three parental lines were grown at Xingtai (XT, 38°N, 115°E), China, in the summer of 2012 using a randomized block design with three replicates. Each plot consisted of 17 individuals grown in a single row with a 5 m length and 0.6 m width. Ten to twelve competitive individuals were harvested from each plot and subsequently air-dried to measure the EL (cm), KNR, ERN and weight per ear including the cob (EW, g).

The 235 NIL-derived F_3_ families were planted in Huanggang (HG), China (18°N, 108°E), in the summer of 2014 using a randomized block design with three replicates. A family was planted in a row containing 13 plants, and 8–10 air-dried ears from a family were used to phenotype EL, KNR, EW and ERN. The average observed value of a given trait across replications was calculated to represent the trait performance for each family.

### Phenotypic data analysis

The phenotypes of four traits in XT and HG were determined by the average of each family from three replicates. The SPSS 20.0 software (http://www.spss.com) was used to calculate the variance components, including the genotype and replication of each trait, using a general linear model. The broad-sense heritability (H^2^) was estimated using the following formula:

$H^{2}\left(\text{\%}\right)=\frac{\sigma_{g}^{2}}{\sigma_{g}^{2}+\frac{\sigma_{e}^{2}}{\text{r}}}\times 100\text{\%}$, where $\sigma_{g}^{2}$ is the genotype variance, $\sigma_{e}^{2}$ is the error variance, and *r* is the number of replicates [17]. The phenotype correlation coefficient was calculated using Pearson correlation analysis.

### Genotyping and QTL mapping

Genomic DNA was extracted using the modified CTAB method. A total of 190 F_2_ individuals of the MT and 269 F_2_ individuals of the WT populations were genotyped using 188 and 176 polymorphic SSR markers, respectively. A genetic map was constructed using the Mapmaker v3.0b software [18]. QTL mapping was performed using WinQTLcart2.5 for composite interval mapping (CIM) [19]. The logarithm of the odds (LOD) score was estimated from 1,000 permutations to determine the presence of a QTL [20]. A QTL explaining more than 10% of the phenotypic variation was considered a major QTL. QTLs detected for different traits with an overlapping confidence interval of 2.0 cM were defined as a QTL cluster [21]. Gene action was characterized as follows: |D/A| = |Dominance effect/Additive effect|, additive (A) 0.00≦|D/A|≦0.20, partial dominance (PD) 0.21≦|D/A|≦0.80, dominance (D) 0.81≦|D/A|≦1.20, and over-dominance (OD) |D/A|>1.21 [22].

To validate *qEL1*.*10*, the genome sequence flanked by BK2N8–umc1862 was retrieved from B73 RefGen V2 to develop new polymorphic markers. In total, 119 SSR markers were used to evaluate the percentage of the recurrent genome of the selected individuals, and sixteen markers including nine newly developed markers (S1 Table) were used to reconstruct the linkage map of the QTL interval.

### Phenotypic evaluation of the hybrids

To evaluate the breeding value of *qEL1*.*10*, six elite inbred lines (Hengbai522, Dong46, Qi319, Yu87-1, H21 and Zheng58) were selected as testers to cross to two QTL-NILs (W138 and W138^TY6^) carrying an allele of *qEL1*.*10* from TY6. A total of 12 hybrids were grown at Huanggang (HG, 18°N, 108°E), China, in the summer of 2015 using a randomized block design with three replicates. Each plot consisted of 22 individuals grown in two rows that were 3 m in length and 0.6 m in width. The ear length (cm), kernel number per row, ear row number and ear weight (g) were measured. The pairwise comparisons between hybrids were conducted using Student's t-test. The general combining ability (GCA) and specific combining ability (SCA) were estimated according to Singh and Chaudhary (1979) [23,24].

## Results

### Phenotype of parental lines and F_2:3_ families

**Fig 1:**
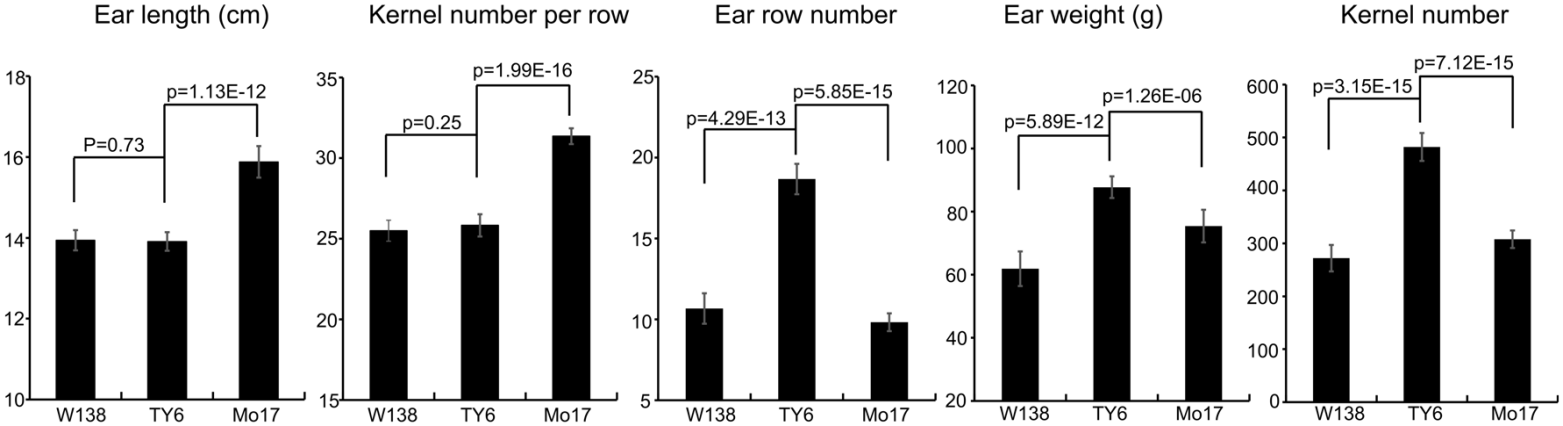
The performance of kernel number-related traits in the three parental lines. EL: ear length; KNR: kernel number per row; ERN: ear row number; EW: ear weight, KN: kernel number.

The phenotypes of the three maize inbred lines used to develop the QTL mapping population were evaluated. Mo17 showed a significantly greater EL (*P* = 1.13E-12) and KNR (*P* = 1.99E-16) than TY6, whereas the EL and KNR of W138 were similar to those of TY6. In contrast, the ERN of TY6 was significantly higher than that of both Mo17 (*P* = 5.85E-15) and W138 (*P* = 4.29E-13). Due to the increased kernel rows on the ears of TY6, TY6 also exhibited a greater kernel number and ear weight relative to Mo17 (*P* = 7.12E-15 and *P* = 1.26E-06, respectively) and W138 (*P* = 3.15E-15 and *P* = 5.89E-12, respectively) (Fig 1).

The phenotypes of the four traits studied in the two mapping populations exhibited normal distributions with obvious transgressive segregation. The analysis of variance showed that the four traits exhibited significant genetic differences among families and high broad-sense heritability that ranged from 75.5% for EW to 88.7% for ERN in the MT F_2:3_ families and from 75.5% for EW to 84.1% for EL in the WT F_2:3_ families. These results indicated that the genetic variance was primarily due to the phenotype variance of these traits in the two populations (Table 1). The correlation analysis showed that EL was highly positively correlated with KNR. Moreover, EL, KNR and ERN were highly correlated with EW (p<0.01). However, ERN was not significantly correlated with EL and KNR in the two populations. These results implied the importance of EL in determining KNR and EW in the two studied populations.

**Table 1:** Phenotypes of four kernel number-related traits in two sets of F_2:3_ families and three parental lines.

Population	Trait	TY6	Mo17	F_2:3_ families					
				Means ± sd	Range	CV(%)	Skew	Kurtosis	H_b_^2^(%)
Mo17×TY6 F_2:3_	EL(cm)	13.9±0.2	15.9±0.4	15.4±1.3	11.1–18.6	8.4	-0.2	0.1	81.8
	KNR	25.8±0.7	31.4±0.5	33.0±3.3	20.0–44.5	10.0	-0.2	1.2	82.3
	ERN	18.7±0.4	9.8±0.6	13.9±1.3	9.9–18.9	9.6	0.2	-0.2	88.7
	EW(g)	87.8±3.4	75.4±5.2	122.0±19.1	60.3–191.9	15.7	-0.2	1.4	75.5
	Trait	TY6	W138	Means ± SD	Range	CV%	Skew	Kurtosis	H_b_^2^ (%)
	EL(cm)	13.9±0.2	13.9±0.3	14.9±1.2	11.4–17.7	8.2	-0.3	-0.3	84.1
	KNR	25.8±0.7	25.5±0.7	30.3±2.9	20.1–37.2	9.6	-0.4	0.2	80.2
	ERN	18.7±0.4	10.7±0.3	14.1±1.5	10.1–20.5	10.8	0.3	0.7	78.6
	EW	87.8±3.4	61.9±5.5	114.9±19.8	55.6–174.8	17.3	0.3	0.3	75.5

EL: ear length; KNR: kernel number per row; ERN: ear row number; EW: ear weight. sd: standard deviation. CV(%): efficient of variation. H_b_^2^(%): broad-sense heritability

### QTL analysis

A total of 188 SSRs in the MT and 176 SSRs in the WT populations were used to construct marker linkage maps across 10 maize chromosomes with a size of 2196.2 cM and 2099.3 cM, respectively. The average intervals between adjacent markers were 11.7 cM in the MT and 11.9 cM in the WT populations. Sequentially, QTL mapping detected sixteen QTLs in the MT and twenty-three QTLs in the WT F_2:3_ families under LOD thresholds that ranged from 2.6 to 3.7 for the different traits studied.

In the MT F_2:3_ families, ten QTLs for EL, KNR and EW were identified. The phenotypic variance explained by a single QTL ranged from 1.9% to 17.6%. *qMEL1*, *qMEL10*, *qMKNR1* and *qMEW8* could explain >10% of the phenotypic variance and were referred to as major QTLs. In these major QTLs (with the exception of *qMKNR1*), increasing alleles were identified in inbred line Mo17. Moreover, six QTLs for ERN were also detected, and the phenotypic variance explained by a single QTL ranged from 1.8% to 15.4%. In total, approximately 37.5% of the QTLs (6/16) were major QTLs and nearly two-thirds of the QTLs (10/16) exhibited partial dominance. Only two QTLs acted in the additive mode (Table 2).

**Table 2:** QTL detected in the two F_2:3_ families.

Population	Trait*	QTL^&^	Marker interval	Range (cM)	Bin	LOD	Genetic effect^$^		R^2^(%)	Gene Action^#^
							Add	Dom		
Mo17×TY6 F_2:3_	EL(cm)	*qMEL1*	ND5~PZE8	213.1~237.3	1.08–1.09	7.8	-0.7	0.4	15.1	PD
		*qMEL2*	umc1026~umc2030	142.3~154.7	2.04	4.5	0.5	0.4	2.8	PD
		*qMEL8*	umc1327~umc1457	4.4~56.1	8.01–8.03	2.8	0.7	0.0	9.9	A
		*qMEL10*	umc1569~umc1556	319.4~342.9	10.07	3.3	0.6	-0.4	15.4	PD
	KNR	*qMKNR1*	ND5~PZE14	213.1~231.6	1.08–1.09	6.5	-1.6	0.7	17.6	PD
		*qMKNR3*	umc1767~umc2152	157.3~201.9	3.08–3.09	3.7	1.6	1.2	3.4	PD
		*qMKNR10*	umc2126~umc1556	328.2~342.9	10.07	3.3	-0.6	1.4	8.7	OD
	EW(g)	*qMEW5*	mmc0481~bnlg1306	135.2~157.9	5.06–5.07	4.3	2.9	10.7	1.9	OD
		*qMEW7*	phi034~bnlg1022	54.8~74.3	7.02	3.6	-6.3	4.0	9.1	PD
		*qMEW8*	umc2212~bnlg1823	91.3~118.3	8.06–8.07	3.0	-1.6	0.7	17.6	PD
	ERN	*qMERN1*	ND18~NND1	139.2~160.2	1.07	8.6	0.6	-0.4	15.4	PD
		*qMERN4*	bnlg1137~umc1194	220.3~234	4.06–4.07	12.4	0.8	0.2	13.5	PD
		*qMERN5-1*	bnlg1046~umc1056	69.1~78.4	5.03	4.2	0.5	0.3	2.6	PD
		*qMERN5-2*	mmc0481~bnlg1306	135.2~157.9	5.06–5.07	7.9	0.7	0.0	9.9	A
		*qMERN6*	umc1805~umc1296	74.8~105.6	6.06	6.1	0.5	0.4	1.8	D
		*qMERN7*	bnlg1022~umc2098	74.3~86.1	7.02	3.8	0.0	0.6	3.2	OD
W138×TY6 F_2:3_	EL(cm)	*qWEL1-1*	HND4~BK2N8	189.6~196.1	1.09	5.5	-0.5	0.2	10.8	PD
		*qWEL1-2*	BK2N1~PZE2	200.4~214.7	1.09–1.10	7.3	-0.6	0.3	15.6	PD
		*qWEL2*	umc2032~umc1065	14.1~34.7	2.04–2.05	8.9	0.6	0.4	6.4	PD
		*qWEL7-1*	umc1718~umc2329	140.3~152.2	7.03	5.3	0.3	-1.8	4.3	OD
		*qWEL7-2*	umc2329~umc2630	152.2~166.4	7.03	5.0	0.3	-2.0	3.5	OD
		*qWEL8*	umc1741~umc1457	42.8~57.5	8.03	4.7	0.2	0.6	0.9	OD
		*qWEL9*	umc1519b~umc1804	131.4~178.6	9.06–9.08	3.8	0.1	0.7	1.0	OD
	KNR	*qWKNR1-1*	HCHR44~HCHR17	35.9~46.8	1.04–1.05	4.3	0.4	1.4	0.4	OD
		*qWKNR1-2*	BK2N1~phi30870	200.4~222.3	1.09–1.10	7.3	-1.5	0.7	17.8	PD
		*qWKNR3*	umc2263~umc1504	56.3~77.1	3.04	3.7	1.0	0.6	2.2	PD
		*qWKNR5-1*	umc1056~umc1990	76.3~100	5.03–5.04	4.6	-0.7	1.1	10.6	OD
		*qWKNR5-2*	umc1990~umc1221	100~118.7	5.04	4.5	-0.7	1.2	9.3	OD
		*qWKNR7*	umc1718~umc2329	140.3~152.2	7.03	5.1	0.3	-5.7	1.1	OD
	EW(g)	*qWEW1*	HND4~PZE2	189.6~214.7	1.08–1.10	5.7	-7.9	3.1	10.4	PD
		*qWEW2*	bnlg2248~umc1065	0~34.7	2.04–2.05	4.2	5.2	6.4	0.8	OD
		*qWEW4*	umc1899~umc2188	197.3~232.8	4.08	7.0	10.2	-1.9	14.4	A
		*qWEW8-1*	umc1457~umc1950	57.5~66.4	8.04	7.3	8.3	7.0	2.3	D
		*qWEW8-2*	umc1950~umc2210	66.4~84.7	8.05	6.5	6.7	7.0	1.6	D
	ERN	*qWERN3*	umc2152~umc2048	294~320.6	3.09	5.4	0.5	0.1	4.5	A
		*qWERN4*	umc1194~umc2188	176.1~232.8	4.07–4.08	22.3	1.2	-0.1	29.5	A
		*qWERN5*	bnlg1879~umc2578	54.7~68.3	5.03	7.8	0.5	0.4	2.3	PD
		*qWERN6*	umc2515~H6CHR10	5.5~15.6	6.01	10.4	0.7	0.2	6.5	PD
		*qWERN9*	umc1519b~umc2359	131.4~203.9	9.06–9.08	3.6	-0.5	-0.1	4.3	A

* Trait. EL: ear length; KNR: kernel number per row; ERN: ear row number; EW: ear weight.

^&^ QTL nomenclature. *qM* represents QTL identified in the Mo17×TY6 F_2:3_ families; *qW* represents QTL identified in the W138×TY6 F_2:3_ families.

^$^ Genetic effect. Add indicates additive effect value; Dom indicates dominant effect value. The values correspond to TY6.

^#^ Gene Action. A: additive; D: dominance; PD: partial dominance; OD: over-dominance.

LOD: logarithm of the odds. R^2^(%): proportion of phenotypic variance explained by single QTL.

In the WT F_2:3_ families, seven QTLs for EL were detected. Of these, *qWEL1-1* and *qWEL1-2* were two major QTLs that acted in the partial dominance mode and separately explained 10.8% and 15.6% of the phenotypic variance, respectively. Six QTLs for KNR were identified on chromosomes 1, 3, 5 and 7; the phenotypic variance explained by a single QTL ranged from 0.4% to 17.8%. Of these QTLs, *qWKNR1-2* and *qWKNR5-1* were major QTLs that exhibited partial dominance and over-dominance, respectively. Of the QTLs for EW, *qWEW1 and qWEW4* explained 10.4% and 14.4% of the phenotypic variance, respectively. Five QTLs for ERN were identified that explained 2.3% to 29.5% of the phenotypic variance. The inbred line TY6 provided increasing alleles for ERN at *qWERN3*, *qWERN4*, *qWERN5*, and *qWERN6* but provided decreasing alleles at *qWERN9*. The *qWERN4* covering known *fea2* [14] of maize showed the largest additive effect and explained approximately 29.51% of the phenotypic variance (Table 2).

By comparing the QTLs detected in the two populations, we found that the allele variations for ERN might occur at multiple loci in the MT population. Therefore, the ERN QTLs accounted for nearly 50% of the QTLs for the four traits detected in the population. In the WT population, each studied trait exhibited allele variations at multiple loci, leading to the identification of multiple QTLs. Additionally, some chromosomal regions were repeatedly covered by overlapping QTLs for different traits, resulting in the formation of five QTL clusters (Table 3). For example, a chromosomal region in bin 1.08–1.10 was covered by *qWEL1-1*, *qWEL1-2*, *qWKNR1-2* and *qWEW1* in the WT population and by *qMEL1 and qMKNR1* in the MT population; these clusters explained 10.4% to 17.8% of the phenotypic variance for different traits (Table 3). The results indicated that the chromosomal region was important for EL, KNR and EW and might be pleiotropic or encode multiple tightly linked genes. This region was designated *qEL1*.*10*.

**Table 3:** QTL clusters detected in the two F_2:3_ families.

cluster	QTL included	Trait	Marker interval	Bin	R^2^(%)
I	*qMEL1*, *qMKNR1*	EL,KNR	ND5~PZE8	1.08–1.10	15.1~17.6
	*qWEL1-1*, *qWEL1-2*, *qWKNR1-2*, *qWEW1*	EL,KNR,EW	HND4~phi30870	1.08–1.10	10.43~17.76
II	*qWEW4*, *qWERN4*	ERN,EW	umc1194~umc2188	4.07–4.08	14.35~29.51
III	*qMEW5*, *qMERN5-2*	ERN,EW	mmc0481~bnlg1306	5.06–5.07	1.9~9.9
IV	*qMEW7*, *qMERN7*	ERN,EW	phi034~umc2098	7.02	3.1~9.1
V	*qMEL10*, *qMKNR10*	EL,KNR	umc1569~umc1556	10.07	8.7~15.4

EL: ear length; KNR: kernel number per row; ERN: ear row number; EW: ear weight. R^2^(%): proportion of phenotypic variance explained by a single QTL.

### Validation of *qEL1*.*10*

**Fig 2:**
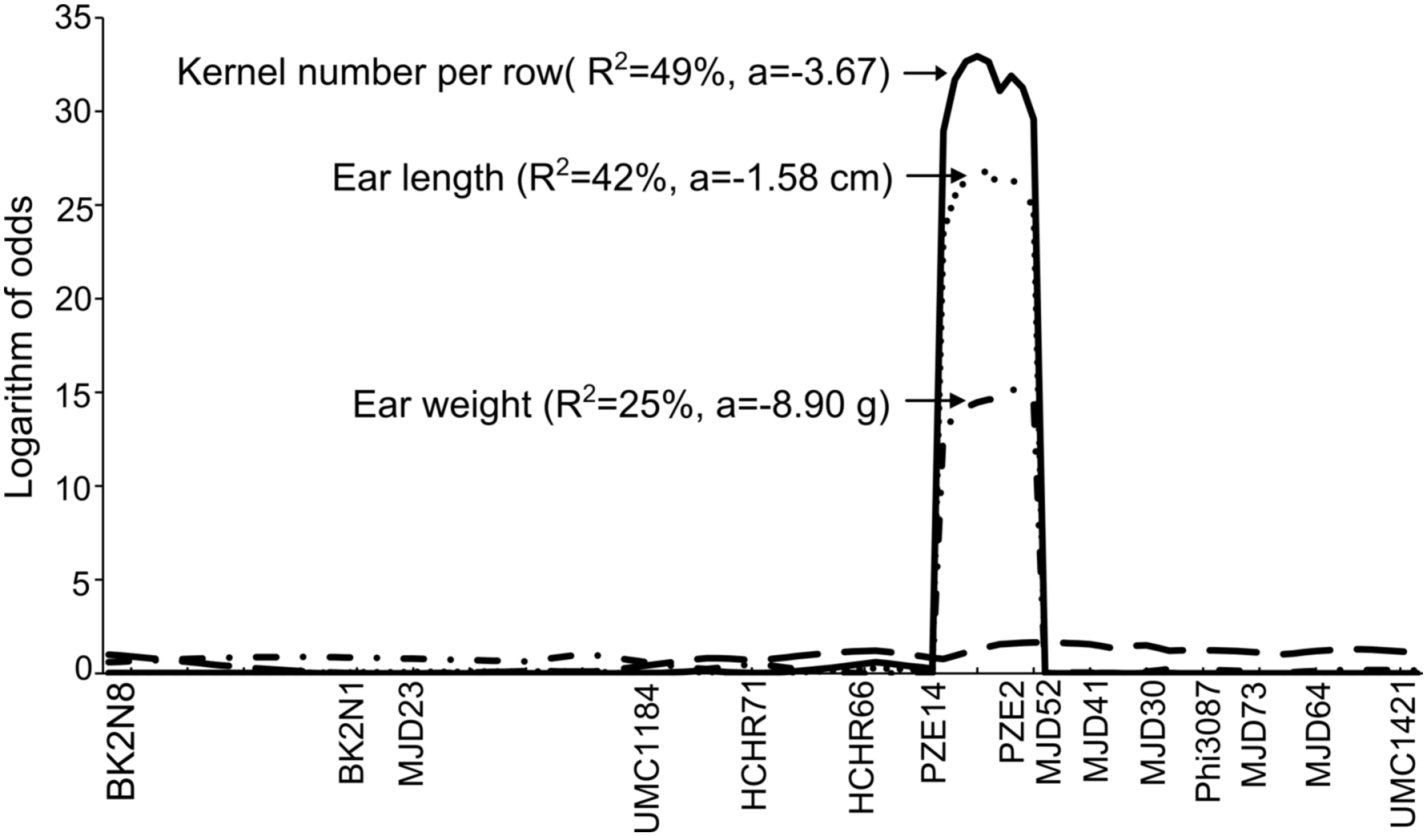
Remapping of *qEL1*.*10* using a near isogenic line-derived mapping population.

To validate *qEL1*.*10*, a BC_4_F_1_ individual with a heterozygous marker genotype within the *qEL1*.*10* interval was chosen and selfed. A line with a homologous marker genotype within the *qEL1*.*10* interval was developed and designated W138^TY6^. Marker screening revealed that the W138^TY6^ line contained 98.1% of the W138 genome. Phenotypic evaluation revealed that the EL (13.2 cm ± 0.4 cm, p = 2.6E-07), KNR (26.1±0.8, p = 2.2E-12) and EW (58.8 g ± 4.5 g, p = 6.2E-06) of W138^TY6^ were significantly lower than those of W138 (Table 4), suggesting the pleiotropism of the substituted chromosome segment. Furthermore, a NIL-derived mapping population with 235 F_3_ families was developed from W138^TY6^×W138. In this population, the *qEL1*.*10* interval was segregating, and four studied traits exhibited high broad-sense heritability ranging from 74.7% for ERN to 83.9% for EL (Table 4). Additionally, EL, KNR and ERN were highly correlated with EW (r>0.5, *p*<0.0001). Sixteen markers including nine newly developed markers were used to reconstruct the linkage map within the *qEL1*.*10* interval. Using QTL IciMapping 3.3, the QTL for EL in BK2N8–umc1862 was detected with a high LOD (26.8) and explained 42% of the EL phenotypic variance. Importantly, we also identified a QTL for KNR within the *qEL1*.*10* interval that explained 49% of the phenotype variance and a QTL for EW that explained 25% of the phenotypic variance simultaneously. The additive effect of the *W138*^*TY6*^ allele was -1.58 cm for EL, -3.67 kernels for KNR and -8.9 g for EW (Fig 2). The QTL interval corresponded to an ~3.0-Mb genomic region in B73 RefGen V2.

**Table 4:** Performance of two inbred lines (W138 and W138^TY6^) and the F_3_ families derived from W138xW138^TY6^.

Trait	Near isogenic lines			F_3_ families					
	W138^TY6^	W138	p-value	Mean ± sd	Range	CV(%)	Skew	Kurtosis	H_b_^2^(%)
EL(cm)	13.2±0.4	14.3±0.5	2.6E-07	16.5±1.2	13.3–18.7	7.8	-0.4	-0.8	89.1
KNR	26.1±0.8	28.9±0.6	2.2E-12	33.2±2.6	26.5–38.3	7.4	-0.4	-0.8	88.5
ERN	10.3±0.7	10.2±0.7	NS	10.0±0.3	9.3–10.9	2.8	0.2	0.2	74.6
EW(g)	58.8±4.5	67.5±4.4	6.2E-06	79.5±8.7	60.6–100.7	10.9	0.4	0.2	83.9

EL: Ear length; KNR: Kernel number per row; ERN: Ear row number; EW: Ear weight. sd: standard deviation. CV(%): coefficient of variation. H_b_^2^(%): Broad-sense heritability. NS: no significant difference at the P < 0.05 level.

Additionally, ear length of W138× the testers, ranging from 16.65 cm to 18.90 cm across all evaluated hybrids, was significantly higher than those of W138^TY6^ × the testers, ranging from 15.79 cm to 17.30 cm. Similarly, KNR and EW of W138 × the testers were also significantly higher than those of W138^TY6^ × the testers (p<0.0001, Student's t-test). However, this phenomenon was not observed for KRN (S2 Table). Because six testers were used to cross to two tested lines (W138 and W138^TY6^), we were able to divide the genetic variance into additive (GCA) and non-additive (SCA) portion. An analysis of variance for the combining ability indicated that GCA variance of the tested lines and SCA variance (tested line × tester) were significant at 1% probability level for ear length, kernel number per row, and ear weight (Table 5). The effect values of the general combining ability of W138 were positive relative to W138^TY6^ (0.76 cm for EL, 1.42 kernels for KNR and 4.31 g for EW)(Table 5 and S2 Table), indicating that the favorable allele of *qEL1*.*10* from W138 could increase the ear length, kernel number per row and ear weight of the hybrid.

**Table 5:** Performance of inbred lines and hybrids and analysis of combining ability.

Type	Genotype	Ear length (cm)	Kernel number per row	Ear row number	Ear weight (g)
Tester	Hengbai522	10.42	18.83	9.37	64.57
	Dong46	12.68	24.49	12.60	80.34
	Qi319	14.42	29.46	11.20	80.80
	Yu87-1	13.76	28.09	11.66	77.58
	H21	11.49	22.14	10.62	64.93
	Zheng58	14.5	24.48	10.33	75.88
Tested line	W138^TY6^	14.3	28.58	10.23	70.86
	W138	13.14	25.90	10.34	62.08
Hybrid	Hengbai522×W138^TY6^	15.86	36.85	10.40	140.70
	Hengbai522×W138	16.65	39.73	10.57	149.10
	Dong46×W138^TY6^	15.79	40.46	13.33	139.30
	Dong46×W138	17.51	43.18	12.67	148.30
	Qi319×W138^TY6^	16.47	43.91	12.67	144.10
	Qi319×W138	17.93	46.17	13.23	145.10
	Yu87-1×W138^TY6^	16.77	43.93	12.83	147.90
	Yu87-1×W138	17.8	46.85	12.62	167.00
	H21×W138^TY6^	16.2	41.54	11.86	138.70
	H21×W138	18.9	45.91	11.64	146.60
	Zheng58×W138^TY6^	17.3	39.58	11.67	126.20
	Zheng58×W138	18.79	41.50	11.40	132.50
	Inbred line mean	13.09	25.25	10.79	72.13
	Hybrid mean	17.16	42.47	12.07	143.79
	Tested line^§^	**(0.76)^#^	**(1.16)	ns	*(4.55)
	Tester	ns^$^	**(0.67)	**(0.89)	*(2.63)
	Tested line × tester	**(0.31)	**(0.47)	ns	**(2.60)

*Significant at the 0.05 probability level.

**Significant at the 0.01 probability level

^#^The data in parentheses represents LSD values for p ≤ 0.05.

^$^ ns, not significant, p > 0.05

^§^ Tested line, tester, and tested line × tester effects represent general combining ability (GCA) for tested line, GCA for tester, and specific combining ability, respectively.

## Discussion

Kernel number, ear row number, ear length and ear weight are widely investigated grain yield-related traits and important maize breeding targets. Hundreds of QTLs for these traits have been detected over the last 30 years using different populations. In particular, several chromosome regions have been repeatedly identified for grain yield-related traits. For example, bin 6.02–6.03 on the maize genome was reported to be a QTL cluster for grain yield, KN, EL and KNR [4, 25,26], and a pleiotropic QTL simultaneously controlling KN, KNR and EL was validated and fine mapped to an approximately 200-Kb region in bin 6.02 [27]. Similarly, a QTL cluster was identified in bin 7.02–7.03 on the maize genome [4,28–30], and the simultaneously pleiotropic QTL *qEL7*.*2* for KNR, EL and EW was validated on bin 7.02 using a NIL-derived population [31]. Additionally, many QTLs for grain yield, KN and ERN have been identified in bin 4.06 and bin 4.08 [12,13]; these two QTL clusters were validated by the isolation of *fea2* [14] and *Ub3* [15] for kernel row number. The results suggest that a QTL cluster for maize yield-related traits is frequently attributed to a pleiotropic QTL for yield components.

In this study, 39 QTLs were detected for the four studied traits in two mapping populations, of which five QTL clusters were detected in both the MT and WT populations. For example, *qMEW7* was co-located with *qMERN7* in bin 7.02 (cluster VI), and *qWEW4* was co-located with *qWERN4* in bin 4.07–4.08 (cluster III). The formation of these QTL clusters is likely a result of the high correlation between the four traits and can be explained by developmental processes: the increase in ear length provides the potential to bear more kernels per ear, which leads to a high grain yield. These QTL clusters have been suggested to be genetically explained by QTL pleiotropy. Moreover, tight linkage of different loci for multiple traits is also a possible contributing factor for these clustered QTLs [32].

Previous studies detected many QTLs for yield-associated traits in bin 1.08–1.10 of the maize chromosome [4,5,33,34]. Intriguingly, in this study we found that six QTLs were co-localized in bin 1.08–1.10 and formed a QTL cluster (cluster I) designated *qEL1*.*10*. *qEL1*.*10* was validated as a simultaneously pleiotropic locus for EL, KNR and EW and was narrowed down to an approximately 3.0-Mb interval (Fig 2). A weak allele of a known mutant may be responsible for the quantitative variation of the corresponding trait; for example, mutant *fea2*, which encodes a leucine-rich repeat receptor-like protein, causes an over-proliferation of the ear inflorescence meristem and a more modest effect on the floral meristem size and organ number in maize [35]. The *fea2* gene was also found to be highly associated with the ear row number of maize [13], and a weak *fea2-1328* allele was validated to increase the number of kernels per row but did not lead to an irregular arrangement of spikelet pair meristems on the female inflorescence [14]. Brown et al. (2011) [13] found that *knotted1* (*kn1*), which is a member of the homeobox gene family [36], was significantly associated with the ear length of maize. In this study, we found that *kn1* was covered by the 3.0-Mb *qEL1*.*10* interval. Therefore, we propose that *kn1* may be responsible for *qEL1*.*10* or may be tightly linkage with the functional locus underlying *qEL1*.*10*.

The high resolution linkage map of bin 1.08–1.10 generated in this study not only contributes to the fine mapping and cloning of *qEL1*.*10* but is also helpful for the improvement of kernel number-related traits by marker-assisted selection. Relative to the allele in TY6, Mo17 and W138 provided increasing alleles at *qEL1*.*10* (Table 2). Therefore desirable alleles at *qEL1*.*10*, such as those in W138 and Mo17, can be used to improve the EL, KNR and EW of hybrids by replacing unfavorable alleles, such as those in TY6 and W138^TY6^.

## Supporting Information

S1 Table Primer sequences of newly developed SSR markers. (DOCX)

S2 Table Performance of hybrids and effect value of the combining ability of two near isogenic lines. (XLSX)
