# Supplementary material for: QTL Mapping of Kernel Number-Related Traits and Validation of One Major QTL for Ear Length in Maize
Source: PLoS One. 2016 May 13;11(5):e0155506. doi: 10.1371/journal.pone.0155506 (PMC4866764; doi:10.1371/journal.pone.0155506)
Supplement: S1 Table — (DOCX) [file pone.0155506.s001.docx]

**S1 Table** Primer sequences of newly developed SSR markers

| Marker name | Forward primer (5′ -3′) | Reverse primer (5′ -3′) |
| --- | --- | --- |
| MJD-23 | TGCTTTCTGTTCCTGCTCCT | CTAGGGACACTAGGCCATGC |
| MJD-30 | GCTCGTTGGGATTTCTTTCA | GGCAAAACCAAAGGTCAAAA |
| MJD-37 | ATGCACAATGCACATGGCTA | GTGCTTCATTGCTTCAGTGG |
| MJD-41 | TGATGATGTCAACGGAAGGA | CCATTTCATCAGTGGCCTGT |
| MJD-52 | AGAACCACCACTGCCAAAAA | GCTCTCATCTTGCCAAAACC |
| MJD-64 | GCCGCTTGTTTCTTTTCCTT | TTCCTTCATGGCTCACCTGT |
| MJD-73 | TGCAAATAAAACCCGGAAAC | GGCCTCCTCTCTCTCTCACA |
| HCHR66 | CTTGCGCCTGCATAGAGAG | AGGTCCTGGCACTAAGAGCA |
| HCHR71 | TGCAGTTCGTACCAGTCAGG | AGCCGGCGAGTCATTAAGAT |
